# Enhancing Legume Ecosystem Services through an Understanding of Plant–Pollinator Interplay

**DOI:** 10.3389/fpls.2016.00333

**Published:** 2016-03-18

**Authors:** María J. Suso, Penelope J. Bebeli, Stefanie Christmann, Célia Mateus, Valeria Negri, Miguel A. A. Pinheiro de Carvalho, Renzo Torricelli, Maria M. Veloso

**Affiliations:** ^1^Department of Plant Breeding, Institute for Sustainable Agriculture, Spanish National Research CouncilCórdoba, Spain; ^2^Laboratory of Plant Breeding and Biometry, Department of Crop Science, Agricultural University of AthensAthens, Greece; ^3^International Center for Agricultural Research in the Dry AreasRabat, Morocco; ^4^Agrarian and Forestry Systems and Plant Protection Unit, National Institute for Agrarian and Veterinarian Research/Linking Landscape, Environment, Agriculture and FoodOeiras, Portugal; ^5^Department of Agricultural, Food and Environmental Sciences, University of PerugiaPerugia, Italy; ^6^ISOPlexis Genebank, University of MadeiraFunchal, Portugal; ^7^Mediterranean Institute of Environmental and Agricultural Sciences, University of EvoraEvora, Portugal; ^8^Biotechnology and Genetic Resources Unit, National Institute for Agrarian and Veterinarian Research/Linking Landscape, Environment, Agriculture and FoodOeiras, Portugal

**Keywords:** heterosis breeding, legume landraces, breeding for seed yield, pest control, Farming with Alternative Pollinators and Crop Design System

## Abstract

Legumes are bee-pollinated, but to a different extent. The importance of the plant–pollinator interplay (PPI), in flowering crops such as legumes lies in a combination of the importance of pollination for the production service and breeding strategies, plus the increasing urgency in mitigating the decline of pollinators through the development and implementation of conservation measures. To realize the full potential of the PPI, a multidisciplinary approach is required. This article assembles an international team of genebank managers, geneticists, plant breeders, experts on environmental governance and agro-ecology, and comprises several sections. The contributions in these sections outline both the state of the art of knowledge in the field and the novel aspects under development, and encompass a range of reviews, opinions and perspectives. The first three sections explore the role of PPI in legume breeding strategies. PPI based approaches to crop improvement can make it possible to adapt and re-design breeding strategies to meet both goals of: (1) optimal productivity, based on an efficient use of pollinators, and (2) biodiversity conservation. The next section deals with entomological aspects and focuses on the protection of the “pest control service” and pollinators in legume crops. The final section addresses general approaches to encourage the synergy between food production and pollination services at farmer field level. Two basic approaches are proposed: (a) Farming with Alternative Pollinators and (b) Crop Design System.

Agricultural land serves first and foremost to produce food and, to an increasing extent, it should contribute to the “green economy” by enhancing diversity in agro-ecosystems. It is therefore often necessary to support farmers in their efforts to improve the biodiversity level on their farms. Legumes are bee pollinated. To realize the full potential of the plant–pollinator interplay (PPI), a multidisciplinary approach is required. The article assembles an international team of genebank managers, geneticists, plant breeders, experts on environmental governance and agro-ecology and comprises several sections. Section “Basic Application-Oriented Questions in Heterosis Breeding: The Potential of Insect-Mediated Outcrossing in Grain Legumes” deals with heterosis breeding strategies. Regarding hybrid and heterotic population breeding, current strategies for using heterosis center on finding ways of reducing the cost and increasing the efficiency of producing hybrid seed. Optimum pollination should be focused on seed production technology based on insect-aided outcrossing. Section “Exploring the Role of Plant–Pollinator Interplay in Grain Legume Landrace Germplasm Conservation and Management” describes landrace pollination aspects from the perspective of *ex situ* and on- farm multiplication. The role played by pollinators can have positive or detrimental effects. While gene flow between different multiplication seed fields can be problematic, insufficient outcrossing within a single population can also create troubles. Data should be available to genebank curators, breeders and farmers to allow the selection of the multiplication strategy in the presence of pollinators and to predict or assess its impact on landrace genetic structure and seed production. Section “Developing Strategies for Seed Production and Seed Producers in Forage Legumes” focuses on strategies for seed production and seed producers in forage legumes. In forage legumes, the potential seed yield (PSY) is very high; many ovules are present per unit area at anthesis, but only a small part of them turns into mature seeds. Seed production is influenced by several factors including the presence of pollinators around seed crop. The section describes general strategies for seed production in forage legumes. Section “Protecting the Pollination Service from Pesticides” brings entomological aspects and focuses on the protection of the “pest control service” and pollinators in legume ecosystems. The use of pesticides to control pests not only affects negatively the pollinators but also the pests’ natural enemies. Enhancing the pest control service of legume ecosystems, by avoiding pesticides and adopting adequate alternative control methods, and by promoting ecological infrastructures, has a positive impact on pollinators. Section “General Approaches to Encourage the Synergy between Food Production and Pollination Services” addresses general approaches to encourage the synergy between food production and pollination services. It is increasingly recognized that pollination has multiple aspects: farmers may use both pollinator-friendly crops and implement practices in the overall production scheme and management of their farms to increase the occurrence, health and visitation of pollinators, whether these are wild or managed. Designing the right crop and identifying the suite of practices, appropriate and effective in a particular site, is where management of pollination becomes key. Two basic approaches are proposed to manage pollination by farmers at field level: (a) Farming with Alternative Pollinators (FAP) and (b) taking into account that flowering crops could be effective determinants of bee diversity and density, the Crop Design System (CDS).

## Basic Application-Oriented Questions in Heterosis Breeding: The Potential of Insect-Mediated Outcrossing in Grain Legumes

### Background

Heterosis (or hybrid vigor) is a natural phenomenon whereby hybrid offspring of genetically diverse individuals display improved physical and functional characteristics relative to their parents (Fu et al., 2014). Heterosis has been increasingly applied in breeding for nearly a century, with the aim of developing more vigorous, higher- yielding, and better-performing cultivars. Hybrid breeding is a success story in several allogamous species such as maize (*Zea mays* L.), sunflower (*Helianthus annuus* L.), and other crops, where, greater yield has been achieved (Coors and Pandey, 1999). Although it is technically more challenging, hybrid breeding is also being approached in partially outcrossing crops such as canola (*Brassica napus* L.) (Longin et al., 2012). Heterosis results in from 20% to over 50% yield increase in inbred species (Tester and Langridge, 2010). For many self-pollinating crop plants, hybrid breeding has been established only recently. The reason for this may lie in the limitations of methods available for controlling pollination and hybrid performance prediction, in particular when established heterotic groups are absent. Strategies for using heterosis more widely to increase yields in inbred crops center on finding ways of reducing the cost and increasing the efficiency of producing hybrid seed (Zhao et al., 2015). In grain legumes, very limited progress has been made in the use of heterosis. So far, hybrid pigeonpea [*Cajanus cajan* (L.) Millsp.] is the only success story to tell in legumes (Palmer et al., 2011).

### Goal and Key Constraints

This section deals with the hybrid breeding technology for the exploitation of heterosis in legumes; it does not review the *status quo* of hybrid breeding, but highlights the main problems currently limiting our capacity to utilize heterotic potential by using the knowledge from the PPI.

The biological constraints of legumes (for more details, see Crop Design System) hamper the implementation of a cost-efficient hybrid seed production technology. The details have been discussed recently (for review, see Palmer et al., 2011; Suso et al., 2015). Briefly, Palmer et al. (2001), listed four components that are crucial for the successful development of hybrids in food legumes: parental combinations that produce heterotic progenies with yield higher than the best pure-line cultivars, a stable male-sterile and female-fertile system, an efficient pollen transfer mechanism from pollen parent to pod parent, and a profitable level of seed increase for the seedsman and grower. However, in legumes, most of the above mentioned components are lacking, making hybrid research an uphill task. Legume hybrid breeding has been less successful, than in other crops because of the lack of knowledge about high-yielding heterotic groups coupled with difficulties in implementing a cost-effective system for hybrid seed production as result of an efficient pollen transfer mechanism from pollen parent to pod parent (Palmer et al., 2011).

Heterotic groups – genotypes or populations – that produce high-yielding progeny after crossing need to be identified. The determination and identification of heterotic combinations or associations require a large number of parental recombinations to be evaluated over a number of years, in many locations, and with an adequate number of replications per location. In legumes, a limiting factor in the study of heterosis is the lack of hybrid seed available for the agronomic performance tests. Most studies were based on a low number of environments with small-sized plots and low seed density (Ortiz-Perez et al., 2007; Palmer et al., 2012).

### Key Examples

Despite these drawbacks, major attempts have been undertaken in the public and private domain to develop hybrid breeding programs in some legumes. Moreover, these attempts at crop improvement programs were recently stimulated by the demand for increased agricultural productivity per area in the face of dynamic environmental and biotic threats which the rapid global environmental changes will make increasingly challenging (Tester and Langridge, 2010). We summarized current knowledge on the extent of hybrid breeding technology through selected examples: a successful story, pigeonpea [*Cajanus cajan* (L.) Millsp], a predominantly selfing crop, soybean [*Glycine max* (L.) Merr.] and a partially allogamous crop, faba bean (*Vicia faba* L.).

#### Pigeonpea

The research by ICRISAT and its collaborators into sterility systems and agronomic performance studies was the catalyst that ensured the success of the pigeonpea hybrid. Saxena et al. (2013) reported that ICRISAT scientists, working with their Indian counterparts, developed the Hybrid ICPH 2671, which showed around 47% superiority for grain yield over the popular cv. control cultivar ‘Maruti’ in multilocation on-station testing and in the on-farm trials. The cytoplasmic-nuclear male sterility, combined with natural outcrossing of the crop (outcrossing was facilitated by a prolonged flowering period) was used to develop viable hybrid breeding technology. Saxena et al. (2013) reported that good amounts of seed set were obtained even with 25–30% outcrossing. The pollen vectors may visit the male-sterile plants several times, and on each visit, a certain proportion of the flowers are pollinated to set the pods, while the un-pollinated flowers should drop. This is followed by the emergence of new flowers on the same plant, some of which again set pods through open pollination. The outstanding performance of this hybrid has led to its release for cultivation in India by both a private seed company and a public university.

#### Soybean

A number of male-sterility systems with appropriate maintainers and restorers are available for soybean hybrid technology. The identification of cytoplasmic-nuclear male-sterile lines along with their maintainers and restorers has been achieved by intraspecific and interspecific hybridizations. Heterosis studies have shown that yield levels above the best parent are possible (Palmer et al., 2011).

Once a stable male-sterility system is obtained, it is necessary to transfer the pollen from the male parent to the female parent. For soybean, a major limitation is the movement of pollen from male parents to female parents. Most of cross-pollinations have been conducted with hand-emasculated plants. Manual cross-pollination to produce large quantities of hybrid seed is difficult and time-consuming. Ortiz-Perez et al. (2006a,b) and Palmer et al. (2009) recommended insect-aided technology for hybrid seed production and considered that the exploitation of the heterosis depends on basic information available on different aspects of the PPI. The role of pollen vectors in soybean production has been generally disregarded, with producers relying on self-pollination and pesticides to maintain yield levels (Milfont et al., 2013). However, growing evidence links improved soybean production to the activity of flower visitors. The studies by Martins (2013), Milfont et al. (2013), and Monasterolo et al. (2015) showed that pollinator activity is important for this crop, leading to increased yield.

Insect-aided crossing potential depends on the functional floral pollinator-related traits of the female and male parents. Studies in cultivated soybean revealed significant differences for floral discovery, attraction and reward traits of the seed parent (Ortiz-Perez et al., 2006a). Ortiz-Perez et al. (2008) indicated that the bees’ preference for certain parental lines was the key factor in reducing hybrid seed production. Among various traits that influence outcrossing, volatiles are crucial. Differences in the emission of flower volatile compounds among soybean cultivars mediate the attractiveness of high and low seed set male-sterility lines. Suso et al. (2010) showed that higher seed setting of male-sterile lines was associated with increases in the length of the standard and changes in its lobe shape. Wild soybean species have a greater ability for outcrossing. *G. syndetika* Pfeil and Craven is a perennial species noted for its relatively intense perfume. In this species, several volatile organic compounds were identified (Palmer et al., 2012; Pappas et al., 2012). Altogether, these results can be exploited to genetically enhance annual soybean cultivars for increased outcrossing.

#### Faba bean

Although good hybrid combinations have been found, none of the many published CMS (cytoplasmic male sterility) systems are employed in practical breeding, mostly due to instability and spontaneous reversion to pollen fertility (for an in-depth review, see Palmer et al., 2011). Hybrid cultivars are not available commercially yet, and are unlikely, due to the high cost of producing and growing very large seeds (Gnanasambandam et al., 2012).

Partially allogamous crops are bred as either self- or cross- fertilizers. Breeding as a self-fertilized crop (line cultivar) leaves heterosis unexploited; moreover, there is a risk of lower yield stability (Maalouf et al., 1999). Genetic uniformity is now being questioned in sustainable agriculture, thus open-pollinated highly heterogeneous and heterozygous populations (mixture or synthetic cultivars) should be an objective in itself (Gasim and Link, 2007; Goldringer et al., 2010).

The heterozygosity level of a population is a function of its outcrossing rate. The expression of heterotic effects in synthetics is particularly dependent on the degree of outcrossing (Maalouf et al., 1999). A prerequisite of breeding synthetic cultivars is the breeding of lines with high cross-fertilization rate (Gasim and Link, 2007).

### Conclusions and Future Perspectives

Hybrids and heterotic populations can contribute to food security through higher yield and resilience. The development of hybrid technology is severely limited, primarily because of its high seed cost due to low outcrossing. Optimization of the insect-aided outcrossing technology is required and should be coupled with the development of a clear understanding of the PPI.

In the context of pollinator decline (Klein et al., 2007), the efficient use of pollinators emerges as a key target (Christmann and Aw-Hassan, 2012; IPBES, 2013; Cost Action FA1307, 2014; Garibaldi et al., 2014). Besides, legume breeding for sustainable agriculture is linked to the development of non-food services such as environmental services (Helenius and Stoddard, 2007; Duc et al., 2014). As it has been the case in other problem-solving matters in plant breeding, the solution lies in the interdisciplinary domain rather than in unique expertise. Structured dialog, as suggested in the framework of the European Innovation Partnerships – Focus Group on Genetic Resources^1^, among pollination biologists, entomologists, as well as breeders, farmers and, beekeepers, offers promising possibilities to overcome the constraint. Significant breakthroughs can be made by mixing disciplines: combining expertise in the core disciplines of agro-ecology, apiculture/entomology, and breeding could result in the development of insect-mediated outcrossing technology.

## Exploring the Role of Plant–Pollinator Interplay in Grain Legume Landrace Germplasm Conservation and Management

### General Introduction to Grain Legume Pollination

In modern agriculture, 19 grain legume crops provide food for mankind, worldwide, or locally (Broughton et al., 2003; Smýkal et al., 2015). Eleven of these crops have records of naturally occurring cross-pollination mediated by PPI (Purseglove, 1968; Klein et al., 2007; Suso et al., 2015). Among these crops are soybean (*G. max*), common bean (*Phaseolus vulgaris* L.), runner bean (*P. coccineus* L.), lima bean (*P. lunatus* Benth.), faba bean (*V. faba* L.), adzuki bean (*Vigna angularis* (Wild.) Ohwi & H. Ohashi), mungo bean [*V. radiata* (L.) R. Wilczek], and cowpea [*V. unguiculata* (L.) Walp.], etc. Many legume crops are considered predominantly self-pollinated (Suso et al., 2011) and their domestication process has favored this pollination behavior (Allard, 1999) with the corresponding adaptation of flower structure and traits (further information is presented in see Crop Design System). Self-pollination has contributed to crop segregation against wild crop relatives (McCouch et al., 2012) and other related legume crops. However, self-pollination can limit the crop diversity and adaptation to the environment, and many crops have kept alternative pollination and reproduction mechanisms to overcome these constrains. Thus, legume self-pollination cannot be seen as an unvarying process (Lloyd and Schoen, 1992), and can be compatible with a variable amount of pollen transfer within the inflorescence or between plants within populations or between populations.

### Assessing Grain Legume Landrace Diversity and Structure

Many grain legumes are traditionally cultivated and maintained worldwide resulting in the evolution of wide genetic diversity represented by landraces (Negri et al., 2013). Genesys (2015) allow us to have an idea of the number of these landraces conserved *ex situ*. There are as many as 75,796 bean landraces, 69.11% of bean germplasm held in genebanks, 19,702 cowpea landraces (63.13%), 31,880 chickpea landraces (57.88%), and 5,754 faba bean landraces (27.95%).

Crop landraces are locally adapted heterogeneous populations and a potential source of genotypes with functional traits needed for PPI and breeding improvement. Landraces have been the matter of widespread analysis but not always of consensual understanding (Zeven, 1998; Pinheiro de Carvalho et al., 2013). However, the importance of landraces, including legume landraces, for crop evolution, food production and agriculture sustainability (Upadhyaya et al., 2011) and eventually for the implementation of the IPM, FAP, and CDS approaches, is widely recognized (see Reducing Pesticide Application by an Integrated Pest Management Approach, Farming with Alternative Pollinators – An Approach to Gain a Mass-Basis for Pollinator Protection, and Crop Design System). Legume landraces are the result of evolutionary adaptation to agro-ecological conditions, and their structure includes the commitment to genetic singularity (assembly of genes and alleles defining the landrace), ecological complexity (defined by environmental adaptation) and socio-cultural value (Brush, 1999; Negri et al., 2009; Freitas et al., 2011).

Legume landraces are submitted to a general trend in modern agriculture characterized by its fast replacement by modern cultivars, with increasing danger of erosion and extinction (Almekinders and de Boef, 1999; Negri et al., 2009; Pinheiro de Carvalho et al., 2016). This trend determines an overall concern for their *ex situ* conservation in germplasm collections, while almost 150 thousands landraces of six major grain legumes are actually stored (see Genesys, 2015).

An important factor for understanding the role of legume landraces in PPI is the knowledge of landrace population structure, in order to comprehend their genetic diversity and the presence of genotypes with required functional traits, and to optimize their *ex situ* and *in situ* (on-farm) conservation and management and/or use in breeding programs. According to our knowledge, the research into this issue has been insufficiently developed. Legume landraces are, by nature, heterogeneous and have a complex structure, whereas traits and genes are evolving in dynamic equilibrium with environmental conditions. The heterogeneity of the landrace can be promoted by farmers in the field through seed exchange, natural or accidental selection and/or gene flow (including that mediated by pollinators through the PPI) within or between closely related landrace populations.

The sampling methodology of landrace material is aimed at gathering as much genetic diversity as possible, while keeping useful landraces traits. If the landrace is strictly self-pollinated, then the seeds harvested will change in terms of the increase of genotypes, leading to a greater fitness in population. In partially cross-pollinated landraces, which allow limited pollen exchange between flowers mediated by pollinators, even small natural outcrossing rates can create many population crosses, since they are composed of several genotypes cultivated in a proximity which allows PPI. In such conditions PPI pollen transfer and allele shuﬄing will increase the heterozygosity of the landrace. Recent data shows that natural outcrossing can contribute to increasing the genetic diversity of crop populations (Papa and Gepts, 2004; Barnaud et al., 2008).

### Impact of Pollinators on the Structure of Grain Legume Landraces: Risks and Opportunities

The impact of pollinators on the heterogeneous structure of landraces is still not well understood. Pollinators can promote self-pollination or outcrossing within each landrace and among landraces or cultivars. Thus, landraces can be vulnerable to changes determined by crosses with other cultivars. Outcrossing with other homogenous cultivars can result in genetic loss of one or several landraces’ useful genotypes and their functional traits. On the other hand, inbreeding depression appears to be related, with genetic erosion and reduction of heterozygosis, when the natural outcross or environmental adaptation are limited. The evolution of the landrace in the field and outcrossing among its genotypes should be facilitated by PPI to avoid inbreeding depression (Street et al., 2008c). Additionally, selfing was found to alter the performance of an open-pollinated population in a progressive way, leading to the expression change of those characters associated with pollinator discovery and attraction (Nadal et al., 2003; Suso and Río, 2014). Martin and Adams (1987), studying the extent of outcrossing in 15 bean landraces in Malawi, identified three different categories regarding their structure. One category was a group of four seed types without evidence of outcrossing among genotypes as result of recent landrace cultivation in the studied region, differences in their flowering times, absence of pollinators activity or pollen incompatibility. In the second category, intermediate outcrossing events between landraces were observed, while in the third category landrace structure had been largely determined by many outcrossing events (Martin and Adams, 1987). These observations point to a temporal change of landrace structure and diversity, promoted by PPI, and need to be taken into consideration, during landrace management.

The global strategy of *ex situ* conservation of legume landraces has resulted in the interruption of evolutionary processes (Brush, 1999). The process can restart periodically, during gene banks management actions, with regeneration or multiplication of accessions. Under cultivation or in on-farm conservation, landraces continue evolving and adapting to environmental conditions. However, this evolution can be threatened by genetic erosion resulting in the extinction of landraces when farmers abandon them or in their genetic pollution by bred cultivars. These cultivars are characterized by genetic gains in yield, but are homogeneous and may not hold the useful traits required for PPI improvement and hybrid seed production (Palmer et al., 2011; see also Key Examples). There are benefits derived from natural outcrossing within landraces for creating new variability and selecting highly adapted yielding cultivars (Saxena and Kumar, 2010). Cross-pollination can also be hazardous for the breeder wishing to maintain the genetic identity and purity of the crop cultivar (Gómez, 2004). The natural outcrossing occurring in several grain legume crops can affect the reliability of the breeding programs when improved cultivars outcross with unimproved plants in nearby fields (Rahman et al., 2001).

The gene flow occurring between landraces and related species depends on outcrossing rates, in addition to the socioeconomic conditions of their cultivation. Oaxacan farmers’ practice multiple landrace cropping systems using common bean, runner bean and *Phaseolus dumosus* across an altitudinal gradient as a strategy to mitigate risks across a broad range of agronomic and household needs that cannot be addressed with single species or races (Worthington et al., 2012). De Mooy et al. (1990) showed that under traditional intercropping systems, outcrossing plays a positive role in maintaining genetic variability and stability of cowpea landraces. However, the outcrossing between landraces and bred cultivars increases the risks of genetic pollution (De Mooy et al., 1990). Additionally, the natural outcrossing in pigeon pea can be considered a constraint for the maintenance of genetic purity of the bred cultivars and genetic seed stocks. New cultivars have been contaminated in a short period of 3–4 years (Gupta et al., 1981). However, Singh et al. (2005) reviewed the long-term Indian pigeon pea breeding program and showed that cultivars were selected from landrace variations created by natural outcrossing, hybridization and segregation events (Singh et al., 1990 cited by Saxena, 2005; Singh et al., 2013).

### *Ex situ* and On-Farm Approaches and the Plant–Pollinator Interplay. A Complex Issue

An important issue in genebank management is to maintain the necessary level of the heterozygosity and heterogeneity of landraces, which can be achieved through the management of pollinator-crop interplay (Suso et al., 2015). Trying to identify gaps in the management of grain legume landraces, Maggioni et al. (2002) analyzed the maintenance and regeneration protocols of 11 germplasm collections and realized that about 50% of them are multiplied under open-pollination conditions. An average contamination rate of 12% for these landraces was estimated, with a high variation between genotype frequency (Maggioni et al., 2002). These data point out the need to focus on collection management protocols, with particular attention to the regeneration procedures and conditions. Recently, recommendations have been drawn up for cowpea, chickpea, faba bean and lentil landrace management (Dumet et al., 2008; Street et al., 2008a,b,c) aiming at supporting the positive results of outcrossing within landraces and to avoid the detrimental effects of cross-pollination with different cultivars.

The Global Crop Diversity Trust has also published regeneration guidelines for many grain legumes, including cultivated chickpea landraces. Even though chickpea (*Cicer arietinum* L.) is mainly self-pollinated, an outcrossing rate of 5.6% has been detected (Rubio et al., 2010), mediated by insects (Purseglove, 1968). An isolation distance of 90 cm between plots has been recommended to avoid unwanted outcrosses (Street et al., 2008c). In pigeonpea, covering the flowering plants with muslin bags or insect-proof cages has been recommended to avoid cross-pollination (Upadhyaya et al., 2008). Regeneration of faba bean landrace germplasm (Street et al., 2008a) can take place in screen houses to avoid insect-facilitated cross-pollination with foreign landraces or cultivars, and it allows within-accession crossing to prevent inbreeding depression. The regeneration in open-pollinated conditions should keep isolation distances to facilitate pollination by natural out crossing within accession.

Self-pollinated *Lens culinaris* Medik. shows a degree of outcrossing, ranging from 0.06 to 5.12%, depending on the cultivar, location, and year (Horneburg, 2006). Additionally, the outcrossing rate between individual flowers in the inflorescence can reach 22.2% within cultivars. Its regeneration will therefore require specific management aimed at controlling pollination, either by allowing insect pollination inside isolation cages or tunnels or in-field free open pollination, keeping the minimum distance between different landraces or cultivars to avoid undesirable outcrosses.

It is vital to maintain landrace integrity, because some genotypes have kept their floral structure, promoting natural outcrosses, which have almost disappeared through crop domestication. At the same time, the genetic mechanisms facilitating the maintenance of outcrossing are relevant in breeding (for both hybrids and populations). Therefore, these flower functional traits will be a useful tool for crop improvement as well as for *in situ* and on-farm conservation (see also Key Examples).

Thus, when regeneration actions are implemented, it is equally important to take notice of pollinators [particularly honey bees (*A. mellifera*), bumble bees (*Bombus* sp.) and hover flies (Syrphidae)] visiting crop flowers, and evaluate the accessions for additional phenological and morphological data. Street et al. (2008b) recommended: (1) coordinating periodic field inspections with entomologists during the growing season, to identify the most efficient visitor/pollinator and determine the relationship between pollinator and flower morphology; (2) evaluating the agronomic data in relation to phenology and flower morphology and structure. Besides, all off-type plants resulting from crossing should be kept and mixed to build a basis population to provide breeders with high-value material (for its outcrossing potential).

## Developing Strategies for Seed Production and Seed Producers in Forage Legumes

### Seed Production in Forage Legumes

#### Genetic and Environmental Factors in Seed Production

Forage legumes show a wide variation in traits influencing breeding programs, cultivar maintenance and seed production. Perennial species are generally cross-pollinated by insects and, consequently, predominantly allogamous. Cultivars of these species (synthetic and improved populations from mass selection or recurrent selection) are populations of different, highly heterozygous genotypes assembled by the breeder in a delicate equilibrium, conditioned by the cross-mating system, that must be maintained during the lifetime of a cultivar, since eventual inbreeding has deleterious effects on forage and seed yields (Ceccarelli, 1986).

Annual medics and subterranean clover are generally self-pollinating species, with cleistogamous flowers and a self-tripping mechanism (Katznelson and Morley, 1965; Lesins and Lesins, 1979). Because of their reproductive system (predominant autogamy) and the huge germplasm variation existing in nature, the breeding of these species has mostly been based on a pure-line selection from natural populations (Piano and Pecetti, 2010).

Seed yield, which is an important factor in determining the success of a cultivar, is determined by genetic and environmental (*sensu lato*) factors. The latter include the choice of the best-suited environment (for soil and climate characteristics) and the choice of an appropriate agronomic technique, where seed sowing rate, care of the establishment (fertilization, irrigation, grazing, and/or cutting management, weeds and pest control) and number of pollinators should be carefully set up. Sub-optimal environmental conditions, cultural and harvesting practices and absence of pollinators negatively condition forage seed yield (Lorenzetti, 1981, 1993; Lorenzetti and Negri, 1995). While research has already clarified the best environmental conditions for seed production (for the main forage species at least), there is still a need to carry out further research on genetic factors affecting seed yield, on which we will focus the following section, with particular reference to minor legumes.

#### Potential Seed Yield vs. Realized Seed Yield

In most forage legumes, the realized seed yield (RSY) is very low compared with the potential seed yield (PSY). The PSY can be estimated multiplying the Potential Seed Number (PSN) per unit area by the average thousand seed weight (TSW) (**Table 1**). The former depends on inflorescence number per unit area, flowers per inflorescence, ovules-per flower (i.e., on the reproductive system size). It is noteworthy that each of these components interacts with the others. With the exception of ovule number per flower, the less variable component, the other components show a high variability in all species (Lorenzetti, 1981; Rosellini et al., 1994), but few data on their hereditability exist. PSY is very high in forages since many ovules are present per unit area at anthesis; however, only a small part of these ovules turns into mature seed and only a part of this seed is collected (**Table 1**). RSY, in fact not only depends on the PSY, but also on the efficiency of the reproductive system (seed setting and maturation) and the percentage of harvested seed.

**Table 1:** Component seed production, potential and realized seed yield of some perennial forage legumes (modified from Lorenzetti, 1993).

Species	Inflorescence (N^o^/m^2^)	Flowers per inflorescence	Ovules per flower	Potential Seed Number/m^2^	Thousand seed weight (TSW) (g)	Potential seed yield (PSY) tha^-1^	Seed harvest realized (RSY)	
							tha^-1^	% of PSY
*M. sativa*	3750	16	10	600,000	2.2	12.0	0.5	4
*T. pratense*	750	110	2	165,000	1.6	2.6	0.5	19
*T. repens*	600	100	6	240,000	0.6	1.8	0.4	22
*L. corniculatus*	400	6	40	96,000	1.2	1.2	0.2	17
*H. coronarium*	2915^∗,∗∗^	30^∗∗∗^	4–5	393,525	4.5	17.7	0.2	1
*O. viciifolia*	2223^∗^	60	1	133,380	18.0	24.0	0.3	1

*^∗^Martiniello (1998); ^∗∗^Satta et al. (2000); ^∗∗∗^Yagoubi and Chrki (2000).*

Pollination and fertilization are essential steps in the formation of mature seeds (Fairey and Hampton, 1998) and mostly depend on pollination. Pollinators like honey bees (*A. mellifera*), leafcutter bees (*Megachile rotundata* Fab.), alkali bees (*Nomia melanderi* Cockerell), and bumble bees (*Bombus* sp.), while foraging, operate flower tripping and fertilization and seed formation occur. Consequently, an adequate number of insects is a key factor in the production of forage seeds. Furthermore, crossing operated by pollinators allows the maintenance of a high level of heterozygosity and heterosis which, as mentioned above, condition important agronomic traits, such as forage and seed yields (Veronesi and Lorenzetti, 1983; Strickler, 1999; Strickler and Vinson, 2000; Pecetti and Proietti, 2003).

The environment strongly influences the success of pollination and fertilization through its effects on flowering pattern, flowering duration, pollen production and vitality, pollen tube growth, stigma receptivity, and ovule viability (Clifford and Scott, 1989; Hampton, 1990; Thomas and Pasumarty, 1996). However, the interplay plant-pollinator also plays a role. For example, in alfalfa, the bee foraging behavior is influenced by flower color (Steiner et al., 1992), flower aroma (Loper et al., 1974), and nectar availability (Kauffeld and Sorensen, 1971; Pecetti and Tava, 2000; Palmer et al., 2009). The latter are partially under genetic control (Loper and Waller, 1970; Loper and Lapioli, 1971; Loper and Berdel, 1978; Pecetti and Tava, 2000; Pecetti et al., 2002) while flower color is totally under genetic control (Barnes, 1966).

Moreover, fertilization may be reduced by genetically determined reproductive barriers such as incompatibility, plant sterility, or unreduced-gametes (Falcinelli, 1999). In addition, a high percentage (50%) of embryo sac degeneration and/or fertilized egg abortion is observed in outcrossing forage legumes (Thomas, 1996) as a possible consequence of the genetic load resulting from deleterious recessive alleles (Wiens, 1984; Burbidge and James, 1991; Pasumarty et al., 1993; Rosellini et al., 1998). Breeding for traits like flower attractiveness to pollinators or low genetic load, factors that indeed condition reproductive efficiency, has been limited up to now.

Poor seed retention of forage legumes is another constraint in achieving PSY, since it causes considerable seed loss before and during harvesting. Forage legumes have not been fully domesticated yet and often show pod dispersal and/or dehiscence of pod prior to harvest. For example, seed shattering coupled with dehiscence of pod is high in *Lotus corniculatus* L., while pod dispersion is high in annual medics, *Trifolium incarnatum* L. and *T. repens*, where losses up to 40% of the total seed yield are reported (Clifford and McCartin, 1985).

### Strategies for Increasing Seed Production in Forage Legumes

Generally, the breeding programs of forage legumes are mainly designed to achieve a balance between persistence, quality, yield, and animal safety (Sewell et al., 2011), while they should also be addressed to increase seed production, since the commercial success of a forage cultivar also depends on its ability to produce seed. High seed yield facilitates the spread of the cultivar because low-cost seed can be easily placed on the market (Lorenzetti and Negri, 1995). In addition, several studies have shown that in forage legumes, seed yield is positively correlated with biomass production (Bolaños-Aguilar et al., 2002; Torricelli et al., 2007).

There are still ample margins for genetic improvement in seed production in forage legumes relying on existing genetic variation for traits that condition SYP (such as inflorescence number and seed number per plant, seed number per inflorescence and seed weight per inflorescence) and there is a high correlation with seed yield per plant (Bolaños-Aguilar et al., 2002; Sengul, 2006), attractiveness for pollinators and seed shattering.

Breeding in these crops is still at an early stage compared with other species. Both improved cultivars and populations are characterized by a wide genetic base, and thus, there is sufficient variation for seed yield to be substantially increased by selection and breeding. Improvements in seed yield will confer substantial benefits to breeding companies and farmers producing forages. It is noteworthy in the context of this paper that cultivars specifically bred for increased attractiveness to pollinators will also help in maintaining the wild pollinator fauna and increase honey production by honeybees (with a consequent increase in economic returns for farmers).

## Protecting The Pollination Service From Pesticides

### The Pest Control Service in Legume Crops

Legume crops are regularly attacked by several pests, pathogens and weeds, at different times of their growth cycles (Stevenson et al., 2007; El-Wakeil and El-Sebai, 2009; Pérez-De-Luque et al., 2010; Sillero et al., 2010; Stoddard et al., 2010; Kharrat and Ouchari, 2011; Stoddard, 2013; Arlauskiene et al., 2015), which strongly affect crop yields worldwide. This frequently compromises the choice of farmers for legume crops (Jensen et al., 2010). These attacks not only affect the crops’ health but also limit the rotation and intercropping schemes that use legumes (Jensen et al., 2010).

Among the biotic constraints of legume crops, insect pests play a major role in yield reduction and several species are reported to be present in legume crops (Stevenson et al., 2007; Stoddard et al., 2010). More than 70 arthropod species have been reported attacking and damaging faba bean (Stoddard et al., 2010). However, not all these enemies are economically important (Nuessly et al., 2004; Stevenson et al., 2007).

The control of pests is crucial to optimizing production; however, frequently, natural enemies are not able to control pest outbreaks efficiently just by themselves in agricultural ecosystems. Presently, the use of pesticides still dominates the pest control programs of legume crops (Stevenson et al., 2007) and natural enemies or introduced (released) ones are chronically exposed to cocktails of pesticides. Predicted changes in global climate are likely to further exacerbate such problems in the future (Goulson et al., 2015).

Pesticides have a negative impact on the pests’ enemies (and also on pollinators, as explained below) by direct mortality and by physiological and behavioral sub-lethal effects. Pesticide effects on their development, adult longevity, immunology, fecundity, sex ratio, mobility, navigation/orientation, feeding behavior, oviposition behavior and learning performance have a great influence in the abundance and pest control performance of those enemies (see details in Desneux et al., 2007). Pesticides disrupt/ weaken the natural pest control service in agricultural ecosystems.

### The Effect of Pesticides on the Pollination Service

Pollination service is another ecological service essential in agricultural ecosystems, for both crops and wild plants. Many insect species contribute to pollination, including bees, hoverflies, butterflies, moths, and some beetles, by visiting a wide or narrow range of flower species to collect food (nectar and/or pollen), thus acting as generalist or specialist pollinators. Bees rely on either nectar or pollen at different stages of their life cycle with a total dependence on flowers, and are considered the most efficient pollinators. European bee species comprise the managed western honey bee (*A. mellifera*), 68 bumble bee species and over 1000 semi-social and solitary bee species (EASAC-The European Academies’ Science Advisory Council, 2015).

There has been a steady decline in many pollinator groups, which has been attributed to several factors, many related to agricultural intensification, such as habitat loss and fragmentation, decreased resource diversity and pesticides application (Steffan-Dewenter et al., 2002; Kremen et al., 2007; Carré et al., 2009; Potts et al., 2010; Brown et al., 2012; Nieto et al., 2014).

Insecticides are the group of pesticides that pose the most direct risk to insect pollinators, especially the neonicotinoids (Woodcock, 2012; Sanchez-Bayo and Goka, 2014; Chagnon et al., 2015; Rundlöf et al., 2015). However, herbicide application also has the indirect effect of damaging the floral resources on which pollinators depend and delaying the flowering, so the timing between the period when food is most needed by pollinators and food availability is disrupted (Boutin et al., 2014; Nieto et al., 2014). Additionally, exposure to some fungicides can greatly increase the toxicity of insecticides, such as neonicotinoids and pyrethroids (Desneux et al., 2007; Goulson et al., 2015).

Pollinators can be exposed to insecticides during their application, by contact with residues, or from the ingestion of contaminated pollen, nectar, or guttation fluid. Exposure occurs in agricultural crops, flowering weedy crop boundaries and also in the neighboring semi-natural habitats where pollinators nest and forage, owing to pesticide drift (Potts et al., 2010). Numerous deaths of pollinators can result from insecticide application to a crop containing blooming weeds, even if the crop itself is not in bloom (Riedl et al., 2006; Gregorc and Ellis, 2011).

Exposure to insecticides may have a lethal effect (e.g., Gradish et al., 2010; Brittain and Potts, 2011; Tapparo et al., 2012; Pisa et al., 2015) or sub-lethal behavioral or physiological effects, such as a reduction in orientation abilities, reduction, or absence of foraging behavior, reduced feeding and life expectancy or compromised pollinators detoxification mechanisms and immune responses, rendering them more susceptible to diseases and parasites (Desneux et al., 2007; Mommaerts et al., 2010; Potts et al., 2010; Brittain and Potts, 2011; Henry et al., 2012; Pettis et al., 2012; Woodcock, 2012; Feltham et al., 2014; Godfray et al., 2014; Goulson et al., 2015).

The life-history traits of pollinator species affect their sensitivity to insecticides, such as body size (contact absorption rate and forage ranges), sociality (colonies essentially bioaccumulate pesticides and social pollinators experience exposure at greater doses for longer periods than solitary ones), flight season (overlapping with pesticide application periods), voltinism (univoltine species foraging when pesticides are applied are at a particular high risk), floral specialization (for polylectic pollinators, sources containing pesticides may be diluted by foraging in other plants or habitats), nesting behavior (ground or above ground nesting may be differently exposed to pesticides), and sex (different intrinsic susceptibility and body size) (see details in Williams et al., 2010; Brittain and Potts, 2011). Although synergistic interactions among traits remain to be explored, individual traits can be useful in predicting and understanding responses of related species to environmental disturbances (Williams et al., 2010).

It must be taken into account that the effects of pesticides on honey bees are poorly representative of the effects on other pollinators, including other bees. Indeed, bees (Apoidea) constitute a highly diverse group, and bees from different taxonomic groups differ widely in their vulnerability to pesticide exposure (Desneux et al., 2007).

In conclusion pollination service benefits from a healthy, robust pest control service in the respective agricultural ecosystem, which reduces the need for artificial pest control such as pesticide application.

### Reducing Pesticide Application by an Integrated Pest Management Approach

The development of pests’ resistance to pesticides, and the economic, health and environmental risks they pose have highlighted the need to reduce pesticide applications (see the European Union Sustainable Use of Pesticides Directive 2009/128/EC) and to adopt alternative pest management methods. To date, no single method provides efficient pest control, and an integrated approach (integrated pest management - IPM) is needed, in which methods are combined in order to maintain enemy populations below their respective economic threshold levels.

The use of pesticides is allowed in the IPM approach, but only when absolutely necessary and after a careful selection (Riedl et al., 2006; Stevenson et al., 2007; Macfadyen et al., 2015). Pest monitoring, together with the concept of economic threshold, allows the perception on when and where to apply pesticides and avoids unnecessary spraying. Removal and protection of bee colonies is also advised (Riedl et al., 2006).

Integrated pest management in legume crops has been widely discussed by several authors (e.g., Holt and Wratten, 1986; Redden et al., 2005; Sharma et al., 2007; Stevenson et al., 2007; Duc et al., 2010; Pérez-De-Luque et al., 2010; Rispail et al., 2010; Sillero et al., 2010; Stoddard, 2013). The wide range of regions where legumes are grown determines a high diversity of crop enemies (Stevenson et al., 2007), and their presence and abundance varies from region to region. Therefore, it is not feasible to define a generalized IPM strategy- it has to be adapted locally or at a regional level.

The biological control method may include releases of artificially reared beneficial organisms; however, a more sustainable way to implement this is to give the natural enemies occurring there the best conditions to increase their density and to enhance their impact on the pests – conservation biological control (CBC). This enhancement of the pest control service is achieved by managing the habitat and manipulating environmental factors (Rebek et al., 2005; Stevenson et al., 2007; Macfadyen et al., 2015).

The success of CBC, and also of the pollination service, is strongly linked to the temporal and spatial availability and quality of ecological infrastructures, inside and outside the farm limits (within a radius of approximately 100–200 m) (Boller et al., 2004), such as shrubs, herbaceous plants (ground covers, inter-row strips, or crop boundaries), wooded field edges, stones, and bare undisturbed soil areas, inside the crop land or in the vicinity (Landis et al., 2000; Westerkamp and Gottsberger, 2000; Richards, 2001; Rebek et al., 2005; Schellhorn et al., 2008; Van Driesche et al., 2008; Köpke and Nemecek, 2010; Shackelford et al., 2013; Sigsgaard et al., 2013). Diverse floral resources (pollen and nectar), with phenological complementarity, apart from their evident importance for pollinators, are of great importance for both predators and parasitoids (Landis et al., 2000; Brown et al., 2012; Döring et al., 2012). When managing existing or introduced floral resources, implementing or preserving different types of shelters, it is essential to know a lot about the ecosystem being manipulated, including the morphology and bio-ecology of the target organisms present there (Landis et al., 2000).

Generally, faba bean offers a rich foraging habitat for several beneficial insects, with mass flowering and substantial amounts of nectar and pollen (Westphal et al., 2003; Nuessly et al., 2004; Köpke and Nemecek, 2010; Stoddard, 2013). For many natural enemies and pollinators, faba bean is considered a substitute for the loss of habitat diversity that results from agricultural intensification (Richards, 2001). Diverse legume leys provide a variety of flowers which bloom sequentially throughout the season to extend the availability of pollen and nectar for pollinators above that of standard leys (Brown et al., 2012).

When adopting IPM and selecting the most suitable pest management methods, it must be taken into account that not only pesticides may be dangerous to natural enemies and pollinators. Cultural practices such as removing weeds, vegetal debris and dead wood with insect galleries, as well as tillage procedures, may have a very negative impact on the beneficial fauna by the destruction of food and refuge sources (Fuchs and Hirnyck, 2000; Riedl et al., 2006; Stevenson et al., 2007; Williams et al., 2010), and so a trade-off of benefits- disadvantages must be considered to reach a correct decision (Saunders et al., 2015).

## General Approaches to Encourage the Synergy Between Food Production and Pollination Services

### Farming with Alternative Pollinators – An Approach to Gain a Mass-Basis for Pollinator Protection

#### The Urgent Need to Get a Mass-Basis for the Protection of Wild Pollinators

The global decline of pollinator diversity is mainly a consequence of human mismanagement (agricultural chemicals, monocultures, landscape fragmentation, habitat loss) as referred to before, and increasingly of different climate change effects and the interaction of these stress factors (Potts et al., 2010). Climate change ultimately requires us to reflect on the PPI from a broader perspective to gain sufficient support for the protection of wild pollinators. Even in the case of agricultural crops, honey bees provide only 8–15% of services (Nabhan and Buchmann, 1997; see also MA, 2005, p. 759).

Pollinators have four key functions within ecosystems and for mankind, which make them indispensable and highlights the need for humans to enhance farming practices and the use of landscapes:

• 87 of the 115 most important food crops depend or benefit from pollination (Klein et al., 2007); this list of high-value crops includes some food legumes such as faba bean, pigeon pea, common bean etc. The total yield, e.g., from faba bean with open access to pollinators is 185% higher compared to autonomous self-pollination (Nayak et al., 2015). This example demonstrates the importance of pollinators for food security and sustainable intensification of agricultural production. Some forage legumes such as alfalfa, clover and vetch depend mainly on wild pollinator species (alfalfa/Megachilidae; clover/*Bombus*);
• 85% of all terrestrial plants require pollinators (Ollerton et al., 2011), and so a loss of pollinators could cause a cascade of extinctions (Biesmeijer et al., 2006) and even pollinator population collapse if stress factors reached a tipping point (Lever et al., 2014). Pollinator protection by sustainable legume production can therefore contribute to the protection of the biodiversity of natural plants;
• Cross-pollination can enhance genetic diversity and thus adaptation to climate change (Christmann and Aw-Hassan, 2012), which is of importance for (1) legume-farmers, e.g., in remote mountainous areas with limited or no access to hybrids, and (2) the much higher number of natural plant species having no human breeders;
• Most other ecosystem services rely on pollination to a certain extent, and the partial, but simultaneous loss of these ecosystem services too could cause a spiral of poverty (Christmann et al., in review).

Cultivated legumes do not have exclusive mutualism between crop and pollinator as, e.g., fig, vanilla, or cocoa, but they are mainly pollinated by wild bees and also by honeybees. Legumes can play an important role in sustaining the diversity of wild bees and some wild bee species are indispensable to sustain some legumes, as the flower type of Fabaceae neither allows all insect pollinators to provide the service nor permits hand-pollination by humans. The extinction of wild bees (leaf cutting bees, solitary bees, or bumblebees) could significantly endanger legume production, in particular faba bean production in temperate or high altitude (above 2500 m) regions and seed production of alfalfa. Faba bean flowers early and faces increasing risks of seasonal abnormalities in the course of climate change, which can prevent honeybees from providing service. Honeybees require 15°C and sunny days without rain and strong wind, whereas the availability of, e.g., bumblebees around the field can safeguard farmers’ revenues despite unfavorable weather conditions.

Wild pollinators mostly work in a small radius (50–2000 m) around their nests (Kohler et al., 2008). Thus, they are more affected by monocultures, ploghing, chemicals, loss of diverse field edges and landscape fragmentation than honeybees, which fly up to 5 km and are supported by beekeepers (transport, food, hives). So the protection of wild pollinators requires a mass-basis of local stakeholders ready to engage themselves in long term pollinator protection. Raising knowledge and awareness of these issues can increase the readiness of smallholders and some benevolent producers to engage in pollinator protection, but the number of commercial producers ready to reduce chemicals and to enhance habitats might be limited, if this action is based on good-will alone (Christmann et al., in review).

### The Methodology of FAP

Hence, the leading research question for FAP (Christmann and Aw-Hassan, 2012) is: how to get a mass-basis of intrinsically motivated local actors? Such a high number of protagonists cannot be paid; even in industrialized countries, the incentive must be grounded on the economic value of ecosystem services (The Economics of Ecosystems and Biodiversity (TEEB)-approach; TEEB, 2010).

Based on broad evidence that a high diversity of pollinators can significantly enhance the harvest of many crops in quantity and quality and that distance between nest and crop is the limiting factor (see literature in Ricketts et al., 2008; Christmann and Aw-Hassan, 2012; Garibaldi et al., 2014), the hypothesis is that by specific crop-based habitat enhancement the total net income from many horticulture crops can be significantly increased; the difference in income between FAP- and control-fields will be sufficient to motivate farmers in favor of pollinator protection by habitat enhancement.

Farming with Alternative Pollinators compares the net revenue of FAP-fields and control fields: whereas control fields have the main crop in the entire field, FAP-fields use only 75% of the field for the main crop and 25% for habitat enhancement, consisting of three-season-forage buffets for pollinators and shelter (both based on further crops, spices, herbs, and medicinal plants), nesting support made out of local materials and a water source, e.g., a puddle or pond. Twelve or more different crops with different flower types and colors nourish pollinators for three seasons. The habitat enhancement in the field itself can optimally reduce the distance between nest and foraging crop. The different flower types and colors of petals attract a higher diversity of pollinators to FAP-fields during the flowering of the main crop. FAP uses a wide range of marketable plants instead of wildflower strips (e.g., (Garibaldi et al., 2014 recommend wildflower strips) to gain the highest possible income gain as an incentive. As the habitat zone and higher insect abundance also attract predators, FAP does not measure the economic value of pollination services, but of habitat enhancement. The methodology for fields and orchards is described in detail in Christmann et al., in review. The options of a purposeful combination of FAP and CBC should be explored and the potential of FAP for enhancing genetic diversity of crops and productivity of seeds should be analyzed. Such supplementary positive impacts on crop productivity by enhanced cross-pollination might additionally motivate farmers to enhance pollinator habitats.

Farming with Alternative Pollinators is a new approach with a limited number of field experiments in Uzbekistan and currently Morocco. The pilot project in Uzbekistan (main crops: cucumber and sour cherry; partly faba bean and partly sainfoin in the habitat zone; Christmann et al., 2014, in review) did on-farm trials mainly with smallholders, because in general they use less or no agricultural chemicals as they prefer low production costs and healthy food for their own families. Trials with smallholders can develop first data to convince commercial farmers too. Starting with large-scale producers can cause discussions about the use of chemicals. Also, due to their high number, smallholders can be important protagonists for pollinator protection.

#### Results of First Field Experiments

The FAP-pilot project confirmed: the FAP-fields attracted a clearly higher number of different pollinator and predator species, including effective wild bee species; for sour cherry, the harvest was double on FAP-sites, for cucumber even higher (Christmann et al., 2014, in review). Solitary bees (important pollinators for cucumber) used the nesting support. Due to the project duration (18 months), it was not possible to assess the adoption rate in the long term, but the readiness to protect pollinators and to take effective action based on data concerning yield increase was much higher in both project regions (Christmann et al., in review) than described in case studies recommending information campaigns (e.g., Kasina et al., 2009a).

#### Potential of FAP with Special Regard to Legumes

Pollinator-friendly legumes can significantly contribute to the habitat zone. Due to their specific flower type and the (for crops) unusual color of the petals, they can attract, e.g., wild bees, which are also effective for many other crops. According to the experience in Uzbekistan it is not difficult to convince farmers to seed faba bean or sainfoin in the habitat zone, as they know they also will benefit directly from these plants. They suggested using crops themselves, and would not consent to plants such as *Laburnum anagyroides* Medik or seed *Indigofera tinctoria* L., ornamental plants or wildflowers (weeds) to attract pollinators (Christmann et al., in review).

Pollinator-dependent legumes might benefit also substantially from FAP as a main crop. Current research on faba bean supports this assumption, since pollinators from nearby natural habitats can increase the harvest significantly (Musallam et al., 2004; Aouar-Sadli et al., 2008; Andersson et al., 2014). Also the production of string bean, soybean, kidney bean, and various peas etc. as main crops, as well as alfalfa-seed production, might benefit from FAP (Klein et al., 2007; referred also in the earlier paragraphs of this article).

As soon as the additional income gain by FAP-measures for these crops as a main crop has been assessed and planting instructions are spread, the intrinsic motivation of farmers for pollinator protection might rise significantly, as the approach does not require investment or long-term training. Including more marketable plants like other crops, spices and medicinal plants, rather than just pollinator-friendly legumes, can provide the necessary mass-basis for pollinator protection. Enhancing the environment might be a better option than the suggestion to breed for pollinator independent crops (Chaplin-Kramer et al., 2014), which has several negative effects: it does not take advantage of heterosis effect and bears the risk of lower yields (see “Basic Application-Oriented Questions in Heterosis Breeding: The Potential of Insect-Mediated Outcrossing in Grain Legumes”), creates the need for continuous breeding to adapt to the changing climate and runs the risk of local pollinator populations starving even more.

### Crop Design System

In the CDS approach, breeders incorporate the potential benefits of pollen vectors into practices to increase the efficiency of hybrid seed technology and, in parallel, increase the occurrence, health and visitation of pollinators, whether these are wild, or managed by developing pollinator-friendly crops.

#### Understanding the CDS Approach

The rationale is the following:

(a) Animal-pollinated crops grown today are often suboptimal in attracting and sustaining their pollinator populations (Bailes et al., 2015). Cultivars are generally bred by the artificial selection of agronomic traits that are of commercial interest but with little regard to pollinator-related traits and preferences. Consequently, insect-pollinated cultivars may not be attractive to pollinators (Kobayashi et al., 2010).

Legumes are known to have flowers with moveable parts that have to be actively handled by insects for pollination to take place. A flower has to be actively moved by a visitor to access the flower reward and, consequently, to perform pollination (Cordoba and Cocucci, 2011). Due to the morphological specialization in floral phenotype, effective pollen transfer requires several traits of the floral mechanism of plants to be adjusted to the morphology and behavior of animal pollen vectors (Garibaldi et al., 2015). Simple morphological mismatches appearing in the flowers are an obvious problem. For instance, hybrid seed production depends on the production and use of male-sterility lines. These lines are difficult to work with naturally in the fields, as the foraging behaviors of bees often change when confronted with such anomalies (Ortiz-Perez et al., 2006b). Information show that some traits may respond to pollinator-driven natural selection (Suso and Río, 2014). Suso and Río (2015) identified some architectural and floral patterns in faba bean that are the result of natural selection and reflect local adaptation to differences in pollination environments. Volatile compounds emitted by crop flowers mediate plant/pollinator interactions (Klatt et al., 2013).

In the CDS approach, breeders and farmers develop cultivars with enhanced heterozygosity and heterogeneity as result of appropriate functional floral traits (discovery, attraction, and reward) within the crop for supporting the bee pollinator populations used as agents of crossings. One idea proposed is to support native pollinators for insect-mediated outcrossing by designing a crop with appropriate functional flower traits (Suso et al., 2005; Palmer et al., 2009; Suso and Maalouf, 2010; Suso et al., 2015).

Legumes have great potential to be served by bee pollinators as crossing agents because they are visited by a great number of bees, such as bumble bees, honey bees, and wild bees (Free, 1993; Delaplane and Mayer, 2000). Non Apis bees, such as solitary bees, are an essential group of visitors and of incalculable value for pollination (e.g., Pierre et al., 1999; Kasina et al., 2009a,b; Suso et al., 2015), even in self-pollinated species such as soybean (Martins, 2013).

However, there is a gap in our knowledge about which particular insects visit and cross-pollinate a particular legume crop, and why. For many legumes, there is little specific data available and even if information on pollinators is available, the findings are often ambiguous (Suso et al., 2015). Besides, the pollinator species can vary from country to country, e.g., faba beans are mainly pollinated by wild bees in Spain and England, but by different species of *Bombus* in France (Bond and Kirby, 1999, 2001; Pierre et al., 1999).

(b) Declines in bee populations have increased interest in the ecological services of legumes (Köpke and Nemecek, 2010). Legume cultivation provides some key environmental benefits such as floral resources. Although the yield produced by legumes remains a major objective for breeders, other issues such as environmentally friendly, resilient production in the context of climate change, as well as new ecological services such as pollinator protection, are required (Duc et al., 2014). Improved value for the conservation of bee pollinators should be a key breeding objective.

#### Concerns and Constrains

Over many centuries, farmers have often selected many legume crops for their autogamous abilities or self-fertility. According to Hammer, (1984 cited by Smýkal et al., 2015), the “domestication syndrome” for legumes includes the transition from outcrossing to selfing. The shift from outcrossing or facultative selfing to strict inbreeding has been described as the single most common trend in legume domestication (Rick, 1988). One common view on the mating system of grain legumes, among germplasm curator and breeders, is that they display autogamous or mostly autogamous mating systems (Suso et al., 2011).

However, for the development of insect-aided outcrossing technology, selfing has to be bred out again and functional floral traits should be managed toward outcrossing. The transition from outbreeding ancestors to autogamously selfing lineages has been derived many times during plant evolution and, in most cases, it is accompanied by a characteristic set of morphological and functional changes to the flowers, together termed the selfing syndrome (Sicard and Lenhard, 2011). The ability of plants to evolve new mating behaviors is a notable feature of plant evolution (Chen et al., 2007).

Legumes possess flowers perfectly capable of outcrossing (Suso et al., 2015). According to Palmer et al. (2009), considering that pollinators respond to differences in discovery, attractiveness and reward, outcrossing could be increased through selection for floral characteristics that enhance its frequency and efficiency. A basic understanding of the variety of mechanisms by which flower morphology and structure influence pollinators’ behavior and consequently pollen transfer, should allow plant breeders to manipulate the PPI in ways that increase insect-mediated outcrossing. Bailes et al. (2015), reviewing key topics in plant-pollinator research, also remark that floral traits are a promising and little-explored avenue for the improvement of crop yields.

Regarding the pollination crisis, breeders should confront the decline of pollinators. One tendency in plant breeding is to opt for plants that do not need pollinators (Partap and Ya, 2012; Chaplin-Kramer et al., 2014). However, yield barriers must be broken. Breaking yield barriers could be achieved by increasing potential yield by improvements in the efficiency of heterosis exploitation (Tester and Langridge, 2010). The clear message is that insect-aided cross-pollination is a key factor in the development of hybrid seed technology and pollinators are essential in providing this service. Changes from a crop that does not require cross-pollination, to crops that respond optimally to the presence of pollen vectors is the key. The proposed breeding strategy is to combine highly self-fertile genotypes which respond optimally to the presence of pollinators to produce high-yielding cultivars. Thus, insect-aided outcrossing allows the exploitation of heterosis potential but, in the absence of pollinators, a minimum yield is achieved (Suso et al., 1996). A delayed selfing mechanism provides reproductive assurance while allowing a high level of outcrossing when pollinators are not a limiting factor (Shirk and Hamrick, 2014).

#### Making the Most of the PPI

In order to maximize pollination success, the employment of new agronomic practices, and the maintenance of semi-natural habitats in the agricultural landscape are essential (Andersson et al., 2014; Nayak et al., 2015; referred to also in section Farming with Alternative Pollinators – An Approach to Gain a Mass-Basis for Pollinator Protection) and should be coupled with the breeding procedure. The CDS approach, considering the management of natural resources, pollen vectors and improving the genetic characteristics of crops for functional floral traits, may prove faster, and more effective. The increasing demands for environmentally friendly agricultural practices and the decline in pollinators have established a favorable context for the development of pollinator-friendly crops, while placing demands on plant breeders to develop cultivars that meet more complex objectives (Duc et al., 2014). But, unfortunately, the analysis of Duc et al. (2014), reflects a relatively small representation of scientific articles related to the hosting of pollinators. Additionally, in spite of the fact that hybrid breeding is considered a breeding technology to increase crop production in a changing world (Tester and Langridge, 2010), the option of considering hybrids and heterotic populations has received minor attention among the breeding approaches. Thus, substantial research is needed to understand better how plants with appropriate functional floral traits could enhance insect-mediated outcrossing and the conservation of pollinator populations, which would help solve practical issues in the development of hybrids and heterotic populations and mitigate pollination declines.

## Author Contributions

All authors contributed to manuscript writing and read and approved the final manuscript. However, regarding that the article include quite different contributions, aspects and disciplines, each author has led and participated mainly in one section, and is going to take responsibility of the particular section. Thus, the individual contributions of each author are being specified. MJS conceived and design the paper and carried out the Section “Basic Application-Oriented Questions in Heterosis Breeding: The Potential of Insect-Mediated Outcrossing in Grain Legumes”. and Crop Design System. PB and MC carried out the Section “Exploring the Role of Plant–Pollinator Interplay in Grain Legume Landrace Germplasm Conservation and Management”. VN and RT carried out the Section “Developing Strategies for Seed Production and Seed Producers in Forage Legumes”. CM and MV carried out Section “Protecting the Pollination Service from Pesticides”. SC carried out section Farming with Alternative Pollinators (FAP) – An Approach to Gain a Mass-Basis for Pollinator Protection.

## Conflict of Interest Statement

The authors declare that the research was conducted in the absence of any commercial or financial relationships that could be construed as a potential conflict of interest.

## Footnotes

1 http://ec.europa.eu/eip/agriculture/en/content/genetic-resources-cooperation-models
